# α-d-Tagatopyran­ose

**DOI:** 10.1107/S1600536809017656

**Published:** 2009-05-23

**Authors:** Francesco Punzo, David J. Watkin, George W. J. Fleet

**Affiliations:** a LAMSUN and CSGI at Dipartimento di Scienze Chimiche, Università degli Studi di Catania, Viale Andrea Doria 6, 95125, Catania, Italy; b University of Oxford, Department of Chemical Crystallography, Chemistry Research Laboratory, Oxford OX1 3TA, England; c University of Oxford, Department of Organic Chemistry, Chemistry Research Laboratory, Oxford OX1 3TA, England

## Abstract

The title compound, C_6_H_12_O_6_, also known as d-Tagatose,  occurs in its furanose and pyranose forms in solution, but only the α-pyran­ose form crystallizes out. In the crystal, the molecules form hydrogen bonded chains propagating in [100] linked by O—H⋯O interactions.  Further O—H⋯O bonds cross-link the chains.

## Related literature

For the d-tagatose market price, syntheses and applications, see: Angyal (1991 ▶); Beadle *et al.* (1992 ▶); Granstrom *et al.* (2004 ▶); Izumori (2002 ▶); Skytte (2002 ▶); Porwell (2007 ▶). For the potential of the title compound as a chiral building block, see: Soengas *et al.* (2005 ▶); Jones *et al.* (2007 ▶, 2008 ▶); Yoshihara *et al.* (2008 ▶). For related crystallographic literature, see: Takagi *et al.* (1969 ▶); Görbitz (1999 ▶); Watkin *et al.* (2005 ▶); Kwiecien *et al.* (2008 ▶); Larson (1970 ▶).
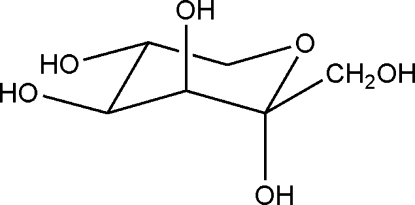


## Experimental

### 

#### Crystal data

C_6_H_12_O_6_
*M* *_r_* = 180.16
Orthorhombic, 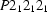

*a* = 6.2201 (1) Å
*b* = 6.5022 (1) Å
*c* = 17.6629 (4) Å
*V* = 714.36 (2) Å^3^
*Z* = 4
Mo *K*α radiation
μ = 0.15 mm^−1^
*T* = 190 K
0.50 × 0.30 × 0.20 mm

#### Data collection

Nonius KappaCCD diffractometer
Absorption correction: multi-scan (*DENZO*/*SCALEPACK*; Otwinowski & Minor, 1997 ▶) *T*_min_ = 0.96, *T*_max_ = 0.97
2343 measured reflections
1378 independent reflections
1351 reflections with *I* > 2.0σ(*I*)
*R*_int_ = 0.010

#### Refinement

*R*[*F* ^2^ > 2σ(*F* ^2^)] = 0.025
*wR*(*F* ^2^) = 0.065
*S* = 0.96
1378 reflections
110 parameters
H-atom parameters constrained
Δρ_max_ = 0.34 e Å^−3^
Δρ_min_ = −0.20 e Å^−3^

### 

Data collection: *COLLECT* (Nonius, 2001 ▶); cell refinement: *DENZO*/*SCALEPACK* (Otwinowski & Minor, 1997 ▶); data reduction: *DENZO*/*SCALEPACK*; program(s) used to solve structure: *SIR92* (Altomare *et al.*, 1994 ▶); program(s) used to refine structure: *CRYSTALS* (Betteridge *et al.*, 2003 ▶); molecular graphics: *CAMERON* (Watkin *et al.*, 1996 ▶); software used to prepare material for publication: *CRYSTALS*.

## Supplementary Material

Crystal structure: contains datablocks global, I. DOI: 10.1107/S1600536809017656/fl2248sup1.cif

Structure factors: contains datablocks I. DOI: 10.1107/S1600536809017656/fl2248Isup2.hkl

Additional supplementary materials:  crystallographic information; 3D view; checkCIF report

## Figures and Tables

**Table 1:** Hydrogen-bond geometry (Å, °)

*D*—H⋯*A*	*D*—H	H⋯*A*	*D*⋯*A*	*D*—H⋯*A*
O4—H41⋯O10^i^	0.81	2.02	2.8236 (14)	171
O9—H91⋯O1^ii^	0.83	1.90	2.7203 (14)	173
O12—H121⋯O4^iii^	0.83	2.09	2.7875 (14)	142
O10—H101⋯O4^iv^	0.81	2.10	2.8518 (14)	155
O1—H11⋯O6^v^	0.81	1.96	2.7661 (14)	175

Symmetry codes: (i) 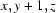; (ii) 
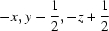
; (iii) 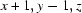; (iv) 
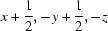
; (v) 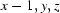.

**Fig. 1.**
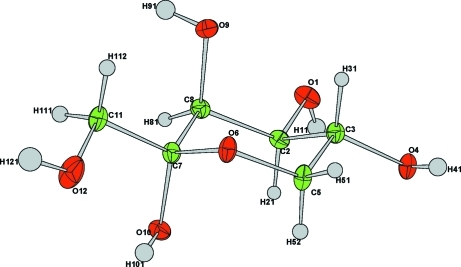
The title compound with displacement ellipsoids drawn at the 50% probability level. H atoms are shown as spheres of arbitary radius.

**Fig. 2.**
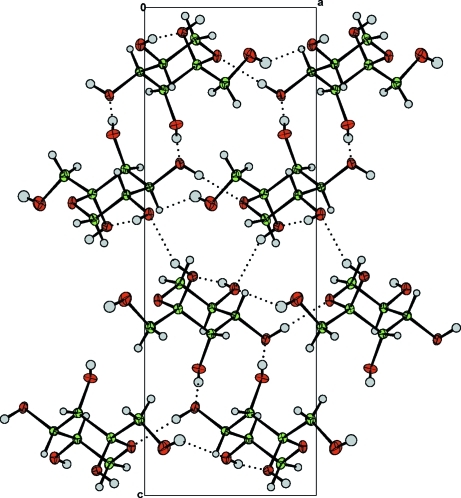
Packing diagram of title compound viewed down the a axis. Hydrogen bonds are shown as dotted lines.

**Fig. 3.**
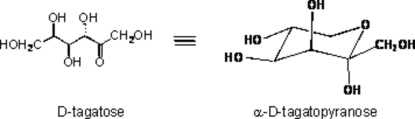
D-Tagatose and α-D-tagatopyranose.

## supplementary crystallographic information

### Comment

Until recently *D*-tagatose was a rare and expensive hexose; the price in the 2007–2008 Aldrich catalogue was 331.00 pounds sterling for 5 g (Porwell, 2007). It is now available cheaply in large quantities [around 5 pounds sterling per kg] prepared by either chemical (Beadle *et al.*, 1992) or biotechnological (Granstrom *et al.*, 2004; Izumori, 2002) techniques, and it is widely investigated as a low calorie sweetener (Skytte, 2002); the potential of *D*-tagatose as a chiral building block is also beginning to be recognized (Soengas *et al.*, 2005; Watkin *et al.*, 2005; Jones *et al.*, 2007; Jones *et al.*, 2008; Yoshihara *et al.*, 2008). The crystal structure of another hitherto rare diasteroisomeric ketohexose, *D*-psicose, has recently been published (Kwiecien *et al.*, 2008). A previous α-*D*-tagatose structure solution (Takagi *et al.*, 1969), did not report either three-dimensional coordinates or bond lengths and angles. Although in aqueous solution both furanose and pyranose forms are present, only the α-pyranose crystallizes out. The crystal structure of the title compound (Fig. 1) consists of a network of hydrogen-bonded chains running parallel to the *a* axis (Fig.2). Referring to Table 1, O4—H41···O10 is the only intramolecular hydrogen bond detected in the structure. O12—H121···O4 and O1—H11···O6 link the molecules into chains, and O9—H91···O1 and O10—H101···O4 stabilize the structure with inter-chain hydrogen bonds. O4 is involved as an acceptor in two hydrogen bonds and as a donor in an almost linear hydrogen bond - the latter by means of H41. The crystal structure shows three equatorial groups and two axial groups, one of which is an axial anomeric hydroxyl group; this would be expected to be the most thermodynamically stable pyranose anomer. The fairly high value of the anisotropic displacement of O12 - compared to the other C and O atoms - is probably due to thermal motion. It results also in a higher - compared to the other H atoms - isotropic displacement for H121 *i.e.* the hydrogen atom connected to the last atom of the flexible C7—C11—O12 chain.

### Experimental

In aqueous solution the major form present is α-D-tagatopyranose (71%) (Fig.1) with 18% of the β-pyranose and small amount of the furanoses (Angyal, 1991). The title compound was recrystallized from a 1:10 mixture of water and acetone allowing the slow competetive evaporation of the solvents, after which, transparent prismatic crystals appeared.

### Refinement

The data were collected with molybdenum radiation and since there were no atoms heavier than Si present, there were no measurable anomalous differences and it was admissible to merge Friedel pairs of reflections. Changes in illuminated volume were kept to a minimum, and were taken into account (Görbitz, 1999) by the multi-scan inter-frame scaling (*DENZO*/*SCALEPACK*, Otwinowski & Minor, 1997). The H atoms were all located in a difference map, but those attached to carbon atoms were repositioned geometrically. The H atoms were initially refined with soft restraints on the bond lengths and angles to regularize their geometry (C—H in the range 0.93–0.98, N—H in the range 0.86–0.89 O—H = 0.82 Å) and *U*_iso_(H) (in the range 1.2–1.5 times *U*_eq_ of the parent atom), after which the positions were refined with riding constraints.

### Crystal data

**Table:**

C_6_H_12_O_6_	*F*(000) = 384
*M**_r_* = 180.16	*D*_x_ = 1.675 Mg m^−^^3^
Orthorhombic, *P*2_1_2_1_2_1_	Mo *K*α radiation, λ = 0.71073 Å
Hall symbol: P 2ac 2ab	Cell parameters from 1344 reflections
*a* = 6.2201 (1) Å	θ = 5–32°
*b* = 6.5022 (1) Å	µ = 0.15 mm^−^^1^
*c* = 17.6629 (4) Å	*T* = 190 K
*V* = 714.36 (2) Å^3^	Prism, colourless
*Z* = 4	0.50 × 0.30 × 0.20 mm

### Data collection

**Table:**

Nonius KappaCCD diffractometer	1351 reflections with *I* > 2.0σ(*I*)
graphite	*R*_int_ = 0.010
ω scans	θ_max_ = 31.5°, θ_min_ = 5.6°
Absorption correction: multi-scan (*DENZO*/*SCALEPACK*; Otwinowski & Minor, 1997)	*h* = −9→9
*T*_min_ = 0.96, *T*_max_ = 0.97	*k* = −9→9
2343 measured reflections	*l* = −25→25
1378 independent reflections	

### Refinement

**Table:**

Refinement on *F*^2^	Hydrogen site location: inferred from neighbouring sites
Least-squares matrix: full	H-atom parameters constrained
*R*[*F*^2^ > 2σ(*F*^2^)] = 0.025	Method = Modified Sheldrick *w* = 1/[σ^2^(*F*^2^) + ( 0.04*P*)^2^ + 0.18*P*], where *P* = (max(*F*_o_^2^,0) + 2*F*_c_^2^)/3
*wR*(*F*^2^) = 0.065	(Δ/σ)_max_ = 0.0001
*S* = 0.97	Δρ_max_ = 0.34 e Å^−^^3^
1378 reflections	Δρ_min_ = −0.20 e Å^−^^3^
110 parameters	Extinction correction: Larson (1970), Equation 22
0 restraints	Extinction coefficient: 260 (40)
Primary atom site location: structure-invariant direct methods	

### Fractional atomic coordinates and isotropic or equivalent isotropic displacement parameters (Å^2^)

**Table:**

	*x*	*y*	*z*	*U*_iso_*/*U*_eq_	
O1	−0.20580 (13)	0.34108 (12)	0.17994 (4)	0.0157	
C2	−0.02883 (15)	0.29006 (15)	0.13171 (5)	0.0113	
C3	0.10689 (16)	0.47890 (15)	0.11569 (5)	0.0118	
O4	−0.02429 (12)	0.62640 (12)	0.07662 (4)	0.0150	
C5	0.29501 (17)	0.41697 (16)	0.06531 (6)	0.0143	
O6	0.42202 (12)	0.25811 (12)	0.09985 (4)	0.0139	
C7	0.30499 (16)	0.07510 (15)	0.11774 (5)	0.0118	
C8	0.11083 (16)	0.12491 (15)	0.16890 (5)	0.0118	
O9	0.18387 (14)	0.19838 (12)	0.24031 (4)	0.0171	
O10	0.22054 (13)	−0.01458 (12)	0.05105 (4)	0.0142	
C11	0.46654 (17)	−0.06848 (16)	0.15537 (6)	0.0155	
O12	0.61351 (15)	−0.12797 (16)	0.09805 (5)	0.0271	
H21	−0.0851	0.2365	0.0849	0.0134*	
H31	0.1585	0.5338	0.1629	0.0151*	
H51	0.3943	0.5322	0.0579	0.0174*	
H52	0.2364	0.3698	0.0173	0.0181*	
H81	0.0263	0.0023	0.1724	0.0147*	
H112	0.5375	−0.0021	0.1979	0.0193*	
H111	0.3929	−0.1876	0.1738	0.0194*	
H41	0.0463	0.7255	0.0642	0.0240*	
H91	0.1860	0.0956	0.2678	0.0275*	
H121	0.7044	−0.2120	0.1130	0.0413*	
H101	0.3225	−0.0416	0.0248	0.0252*	
H11	−0.3163	0.3242	0.1565	0.0238*	

### Atomic displacement parameters (Å^2^)

**Table:**

	*U*^11^	*U*^22^	*U*^33^	*U*^12^	*U*^13^	*U*^23^
O1	0.0103 (3)	0.0192 (4)	0.0177 (3)	−0.0009 (3)	0.0026 (3)	−0.0047 (3)
C2	0.0102 (4)	0.0120 (4)	0.0118 (4)	−0.0005 (4)	0.0005 (3)	−0.0019 (3)
C3	0.0107 (4)	0.0107 (4)	0.0141 (4)	0.0001 (3)	−0.0003 (3)	0.0004 (3)
O4	0.0131 (3)	0.0119 (3)	0.0201 (3)	0.0026 (3)	−0.0007 (3)	0.0027 (3)
C5	0.0125 (4)	0.0121 (4)	0.0183 (4)	0.0022 (4)	0.0034 (4)	0.0041 (3)
O6	0.0097 (3)	0.0111 (3)	0.0209 (3)	−0.0001 (3)	−0.0007 (3)	0.0036 (3)
C7	0.0112 (4)	0.0100 (4)	0.0143 (4)	−0.0003 (4)	−0.0007 (3)	0.0007 (3)
C8	0.0124 (4)	0.0107 (4)	0.0122 (4)	−0.0020 (4)	−0.0001 (3)	−0.0004 (3)
O9	0.0243 (4)	0.0154 (3)	0.0115 (3)	−0.0007 (3)	−0.0033 (3)	0.0001 (3)
O10	0.0138 (3)	0.0151 (3)	0.0137 (3)	0.0009 (3)	0.0005 (3)	−0.0030 (3)
C11	0.0136 (4)	0.0139 (4)	0.0191 (4)	0.0026 (4)	−0.0015 (4)	0.0033 (4)
O12	0.0213 (4)	0.0322 (5)	0.0280 (4)	0.0161 (4)	0.0044 (4)	0.0078 (4)

### Geometric parameters (Å, °)

**Table:**

O1—C2	1.4309 (12)	O6—C7	1.4303 (12)
O1—H11	0.810	C7—C8	1.5426 (14)
C2—C3	1.5167 (14)	C7—O10	1.4155 (12)
C2—C8	1.5294 (14)	C7—C11	1.5241 (14)
C2—H21	0.963	C8—O9	1.4232 (11)
C3—O4	1.4359 (12)	C8—H81	0.957
C3—C5	1.5241 (14)	O9—H91	0.826
C3—H31	0.963	O10—H101	0.805
O4—H41	0.810	C11—O12	1.4178 (14)
C5—O6	1.4364 (12)	C11—H112	0.973
C5—H51	0.980	C11—H111	0.957
C5—H52	0.972	O12—H121	0.829
			
C2—O1—H11	108.5	O6—C7—C8	110.68 (8)
O1—C2—C3	110.58 (8)	O6—C7—O10	110.35 (8)
O1—C2—C8	110.13 (8)	C8—C7—O10	106.46 (8)
C3—C2—C8	109.41 (8)	O6—C7—C11	105.69 (8)
O1—C2—H21	108.4	C8—C7—C11	112.92 (8)
C3—C2—H21	109.6	O10—C7—C11	110.81 (8)
C8—C2—H21	108.7	C7—C8—C2	109.91 (8)
C2—C3—O4	108.31 (8)	C7—C8—O9	109.85 (8)
C2—C3—C5	108.81 (8)	C2—C8—O9	109.04 (8)
O4—C3—C5	109.40 (8)	C7—C8—H81	107.0
C2—C3—H31	108.9	C2—C8—H81	107.5
O4—C3—H31	111.0	O9—C8—H81	113.5
C5—C3—H31	110.3	C8—O9—H91	104.7
C3—O4—H41	110.7	C7—O10—H101	106.1
C3—C5—O6	111.36 (8)	C7—C11—O12	106.31 (8)
C3—C5—H51	111.1	C7—C11—H112	111.4
O6—C5—H51	105.1	O12—C11—H112	112.4
C3—C5—H52	107.7	C7—C11—H111	109.2
O6—C5—H52	110.4	O12—C11—H111	109.3
H51—C5—H52	111.2	H112—C11—H111	108.3
C5—O6—C7	114.34 (8)	C11—O12—H121	113.0

### Hydrogen-bond geometry (Å, °)

**Table:**

*D*—H···*A*	*D*—H	H···*A*	*D*···*A*	*D*—H···*A*
O4—H41···O10^i^	0.81	2.02	2.8236 (14)	171
O9—H91···O1^ii^	0.83	1.90	2.7203 (14)	173
O12—H121···O4^iii^	0.83	2.09	2.7875 (14)	142
O10—H101···O4^iv^	0.81	2.10	2.8518 (14)	155
O1—H11···O6^v^	0.81	1.96	2.7661 (14)	175

Symmetry codes: (i) *x*, *y*+1, *z*; (ii) −*x*, *y*−1/2, −*z*+1/2; (iii) *x*+1, *y*−1, *z*; (iv) *x*+1/2, −*y*+1/2, −*z*; (v) *x*−1, *y*, *z*.

## References

[bb1] Altomare, A., Cascarano, G., Giacovazzo, C., Guagliardi, A., Burla, M. C., Polidori, G. & Camalli, M. (1994). *J. Appl. Cryst.* **27**, 435.

[bb2] Angyal, S. J. (1991). *Adv. Carbohydr. Chem. Biochem.* **49**, 19–35.

[bb3] Beadle, J. R., Saunders, J. P. & Wajda, T. J. (1992). Process for Manufacturing tagatose, US Patent 5 078 796, January 7, 1992.

[bb4] Betteridge, P. W., Carruthers, J. R., Cooper, R. I., Prout, K. & Watkin, D. J. (2003). *J. Appl. Cryst.* **36**, 1487.

[bb5] Görbitz, C. H. (1999). *Acta Cryst.* B**55**, 1090–1098. 10.1107/s0108768199008721 10927450

[bb6] Granstrom, T. B., Takata, G., Tokuda, M. & Izumori, K. (2004). *J. Biosci. Bioeng.* **97**, 89–94. 10.1016/S1389-1723(04)70173-5 16233597

[bb7] Izumori, K. (2002). *Naturwissennshaften*, **89**, 120-124.

[bb8] Jones, N. A., Jenkinson, S. F., Soengas, R., Fanefjord, M., Wormald, M. R., Dwek, R. A., Kiran, G. P., Devendar, R., Takata, G., Morimoto, K., Izumori, K. & Fleet, G. W. J. (2007). *Tetrahedron Asymmetry*, **18**, 774–786.

[bb9] Jones, N. A., Rao, D., Yoshihara, A., Gullapalli, P., Morimoto, K., Takata, G., Hunter, S. J., Wormald, M. R., Dwek, R. A., Izumori, K. & Fleet, G. W. J. (2008). *Tetrahedron Asymmetry*, **19**, 1904–1918.

[bb10] Kwiecien, A., Slepokura, K. & Lis, T. (2008). *Carbohydrate Res.* **343**, 2336–2339. 10.1016/j.carres.2008.05.012 18547550

[bb11] Larson, A. C. (1970). *Crystallographic Computing*, edited by F. R. Ahmed, S. R. Hall & C. P. Huber, pp. 291–294. Copenhagen: Munksgaard.

[bb12] Nonius (2001). *COLLECT* Nonius BV, Delft, The Netherlands.

[bb13] Otwinowski, Z. & Minor, W. (1997). *Methods in Enzymology*, Vol. 276, *Macromolecular Crystallography*, Part A, edited by C. W. Carter Jr & R. M. Sweet, pp. 307–326. New York: Academic Press.

[bb14] Porwell, J. (2007). *Aldrich Handbook of Fine Chemicals* p. 2253. Milwaukee, WI, USA: Aldrich.

[bb15] Skytte, U. P. (2002). *Cereal Foods World*, **47**, 224–227.

[bb16] Soengas, R., Izumori, K., Simone, M. I., Watkin, D. J., Skytte, U. P., Soetaert, W. & Fleet, G. W. J. (2005). *Tetrahedron Lett.* **46**, 5755–5759.

[bb17] Takagi, S. & Rosenstein, R. D. (1969). *Carbohydrate Res.* **11**, 156–158.

[bb18] Watkin, D. J., Glawar, A. F. G., Soengas, R., Skytte, U. P., Wormald, M. R., Dwek, R. A. & Fleet, G. W. J. (2005). *Acta Cryst.* E**61**, o2891–o2893.

[bb19] Watkin, D. J., Prout, C. K. & Pearce, L. J. (1996). *CAMERON*, Chemical Crystallography Laboratory, Oxford, UK.

[bb20] Yoshihara, A., Haraguchi, S., Gullapalli, P., Rao, D., Morimoto, K., Takata, G., Jones, N. A., Jenkinson, S. F., Wormald, M. R., Dwek, R. A., Fleet, G. W. J. & Izumori, K. (2008). *Tetrahedron Asymmetry*, **19**, 739–745.

